# Interplay of miRNAs and lncRNAs in STAT3 signaling pathway in colorectal cancer progression

**DOI:** 10.1186/s12935-023-03202-3

**Published:** 2024-01-07

**Authors:** Omid Rahbar Farzam, Souzan Najafi, Mohammad Amini, Zohreh Rahimi, Reza Dabbaghipour, Omid Zohdi, Ghazale Asemani Shahgoli, Behzad Baradaran, Bahman Akbari

**Affiliations:** 1 Department of Medical Biotechnology, School of Medicine, Kermanshah University of Medical Sciences, Kermanshah, Iran; 2 Immunology Research Center, Tabriz University of Medical Sciences, Tabriz, Iran; 3 Department of Clinical Biochemistry, Medical School, Daneshgah Avenue, Kermanshah, Iran; 4 Medical Biology Research Center, Daneshgah Avenue, Kermanshah, Iran; 5 Department of Medical Genetics, Shiraz University of Medical Sciences, Shiraz, Iran; 6 Medical Biology Research Center, Kermanshah University of Medical Sciences, Kermanshah, Iran

**Keywords:** Non-coding RNA, miRNA, lncRNA, STAT3, Colorectal cancer, Review, Treatment, Tumorogenesis, Cancer

## Abstract

In recent decades, colorectal cancer (CRC) has turned into one of the most widespread malignancies, and the incidence of this malignancy is expected to increase. Despite considerable improvements in therapeutic approaches, the prognosis, and the management of CRC face many problems. Likely, the main limitation in the successful treatment of CRC is the lack of appropriate clinical therapeutic targets. As an effective target, the signal transducer and activator of transcription 3 (STAT3) are regulated by a wide range of genes and involved in cellular processes, including cell growth, migration, invasion, immunosuppression, and angiogenesis. Aberrant regulation of STAT3 signaling leads to cellular dysfunction, diseases, and malignancies, including CRC. Consequently, targeting this signaling pathway is considered one of the therapeutic strategies used in CRC treatment. MicroRNAs (miRNAs) and long non-coding RNAs (lncRNAs) are non-coding RNA molecules with partial or no protein-coding activity that participate in gene regulation at epigenetic, transcriptional, and post-transcriptional levels and regulate multiple signaling pathways, including STAT3 signaling (especially JAK/STAT). Therefore, these regulatory molecules are suggested to be very promising targets to present new insights into overcoming the limitations of conventional therapeutic strategies. Therefore, the current review study aimed to summarize the therapeutic and diagnostic significance of miRNAs and lncRNAs and their therapeutic and diagnostic significance related to the expression and activity of STAT3 in CRC.

## Introduction

Cancer is one of the leading causes of death in societies today [1], and one of the most important types is colorectal cancer (CRC). CRC is considered one of the most frequent types of gastrointestinal cancers, ranked the third most widespread malignancy among all types of cancers, and the second deadliest malignancy globally, with 1,931,590 new cases and more than 900,000 deaths in 2020 [2]. The CRC incidence was predicted to rise to 60% by 2030 and is more likely to be diagnosed in men than women [3]. CRC includes two general types of colon cancer (CC) and rectal cancer (RC) [4]. Although CRC incidence involves the statistics of both malignancies, RC accounts for 28% of all patients, whereas CC accounts for 72% of new cases [2].

CRC is characterized by the transformation of the healthy colon and rectum epithelium into a pre-cancerous lesion, known as adenomatous intermediate, which ultimately turns into the adenocarcinoma, an invasive form of carcinoma, which can migrate to distinct organs and results in metastatic damage, especially in the liver [5]. This process involves the activation of oncogenes and inactivation of tumor suppressor genes caused by epigenetic alterations and genetic mutations. The epigenetic mechanisms include histone modification, DNA methylation, and non-coding RNAs (ncRNAs), discovered throughout 10 to 15 years. These mechanisms may represent a distinct pathology, affect individual susceptibility to cancer, and potentially participate in the appearance of resistance to therapeutic methods in CRC patients [5–7].

In the CRC study, non-coding RNAs (ncRNAs) have gained growing importance, significantly altering our understanding of the molecular basis of this disease [8]. Although protein-coding genes have historically received more attention, ncRNAs, which do not encode proteins, have emerged as significant players in the initiation, development, and metastasis of CRC due to their wide range of essential functions [9]. MicroRNAs (miRNAs) and long non-coding RNAs (lncRNAs) are two types of ncRNAs that have gained significant attention within the field of cancer research, particularly about CRC [10]. These ncRNAs have been recognized for their capacity to modulate crucial cellular processes and signaling pathways that play a pivotal role in the development and progression of CRC [11].

CRC has a complex pathogenesis involving many factors, and one of the key contributors is the signal transducer and activator of transcription 3 (STAT3) signaling pathway [12]. STAT3 is a transcription factor extensively linked to the onset and progression of CRC [11, 12]. It plays a crucial role in regulating inflammatory processes in colitis-associated cancer and exhibits heightened activation in CRC, hence facilitating the proliferation of cancer cells, boosting tumor growth, stimulating angiogenesis, and facilitating invasion and migration [13, 14]. In addition, the emerging domain of ncRNA investigation has revealed the intricate regulatory functions of miRNAs and lncRNAs in CRC [15]. NcRNAs play a vital role in regulating the activity of STAT3 [8, 9, 12]. This introduces an additional level of intricacy to the complicated network involved in CRC evolution. miRNAs exert significant regulatory control over gene expression within the STAT3 pathway by modulating gene activity at the post-transcriptional level. Consequently, this phenomenon directly impacts the complex balance of CRC progression [16]. Concurrently, lncRNAs have become exciting players in this field, with various roles like binding to STAT3, affecting its phosphorylation, or indirectly controlling the genes it regulates [17]. Collectively, these actions contribute to the overall landscape of CRC [16, 17].

Understanding how STAT3 interacts with these ncRNAs is crucial for unraveling the complex molecular mechanisms behind CRC and finding new ways to treat it. This review aims to explore the importance of ncRNAs like lncRNAs and miRNAs in the development of CRC through the STAT3 pathway, which is a critical factor in the initiation and progression of CRC.

## Overview of non-coding RNAs

**Fig. 1:**
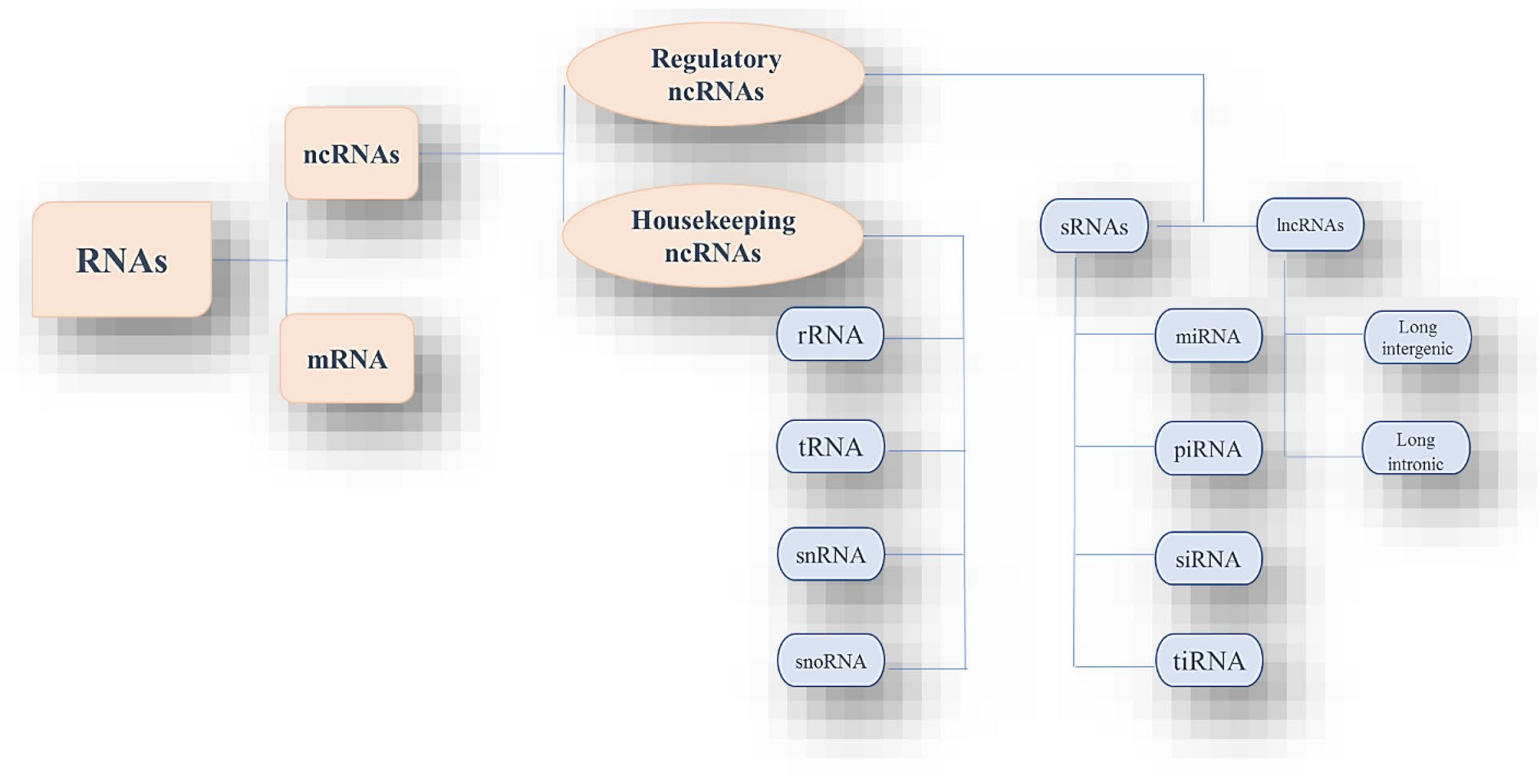
The classification of RNAs and non-coding RNAs

Comprehensive genome sequencing has illustrated that over 90% of the human genome is transcribed, but approximately 20,000 protein-coding RNA are encoded, composing less than 2% of the whole genome [18]. More studies have discovered many ncRNA species, which are suggested to play crucial roles in numerous biological activities through direct or indirect interference with gene expression [19]. In addition to well-known ncRNAs like tRNA and rRNA involved in protein synthesis, ncRNAs encompass novel transcripts. As shown in Fig. 1, the classification of RNAs and ncRNAs, we can categorize ncRNAs into two classes: housekeeping ncRNAs (e.g., rRNAs, tRNAs, snRNA, snoRNAs) and regulatory ncRNAs (e.g., miRNAs, piRNAs, siRNAs, lncRNAs, tiRNAs) [7]. Housekeeping ncRNAs are abundant and ubiquitous, vital for fundamental cellular functions. On the other hand, regulatory ncRNAs play critical roles in gene regulation across various levels (epigenetic, transcriptional, post-transcriptional) and can be classified into small ncRNAs (e.g., miRNA, siRNAs, piRNA) with sizes usually below 200 nucleotides, and long ncRNAs (typically exceeding 200 nucleotides), including long intronic ncRNAs and long intergenic ncRNAs within this regulatory ncRNA category [20–22].

MiRNAs, major small ncRNAs, are endogenous, single-stranded, and highly conserved molecules, typically 21–23 nucleotides long, derived from transcribed hairpin loop structures [23]. The maturation process of miRNAs involves nuclear and cytoplasmic stages. In the nucleus, RNA polymerase II transcribes most miRNAs from DNA into primary miRNA precursors (pri-miRNAs), which acquire a 5’ cap and poly-A tail in their 3’ UTRs. RNase III processes pri-miRNAs into miRNA stem-loop precursors (pre-miRNAs) [7]. Upon translocation to the cytoplasm and subsequent enzymatic activities, one strand of pre-miRNAs fulfills their regulatory function through the RNA-induced silencing complex (RISC). MiRNAs, in conjunction with RISC, bind to the 3’-UTR of target RNA sequences, leading to mRNA cleavage or inhibition of translation. This regulation impacts biological processes such as cell growth, cell cycle distribution, differentiation, apoptosis, invasion, epithelial-mesenchymal transition (EMT), and migration. Recent research indicates that miRNAs can bind not only to the 3’-UTR but also to the 5’-UTR and promoter regions of target genes. Unlike 3’-UTR binding, miRNA binding to the 5’-UTR stabilizes the target mRNA or enhances its translation or transcription [24]. Also, in recipient cells, miRNAs may be secreted and modulate the expression of the target gene via the exosomal pathway [25]. In various cancers, including CRC, miRNAs often display dysregulated expression, functioning as either oncogenes (oncomiRs) or tumor suppressors, impacting cancer initiation and progression [26]. Additionally, due to their structural stability, miRNAs hold significant potential as diagnostic and prognostic molecular markers in various human cancers, including CRC.

Among ncRNAs, lncRNAs comprise the most widespread and functionally varied class [27]. However, over the past decade, there has been uncertainty regarding lncRNA function, as they typically do not encode proteins, though some may produce small peptides [28]. Most lncRNAs originate from the antisense regions upstream of promoters. RNA polymerase II is responsible for their transcription, resulting in lncRNAs having cap structures and poly-A tails, and they also undergo multiple splicing modifications in the nucleus. Additionally, other mechanisms may be involved in lncRNA maturation [29]. LncRNAs are classified into five subgroups based on their genomic location and relationship to protein-coding genes, including long intergenic ncRNAs (lincRNAs), long intronic ncRNAs, sense lncRNAs, antisense lncRNAs, and bidirectional lncRNAs [30]. LincRNAs originate from both strands in intergenic areas, while long intronic ncRNAs are entirely transcribed from gene introns. Sense lncRNAs are transcribed from the sense strand within exonic regions of protein-coding genes, whereas antisense lncRNAs are transcribed from the antisense strand. Bidirectional lncRNAs are located near coding transcripts on the opposing strand [31]. Regarding their regulatory influence on DNA sequences, lncRNAs are categorized as cis-lncRNAs, which regulate gene expression nearby, and trans-lncRNAs, which govern distant gene activity [32]. LncRNAs serve various roles, including as decoys (e.g., PANDAR and MALAT1) preventing target binding to chromatin, guides (e.g., Xist, HOTAIR, COLDAIR) directing chromatin-modifying enzymes, signals (e.g., lincRNA-p21, COLDAIR, HOTAIR) mediating transcription processes, and scaffolds (e.g., HOTAIR, ANRIL) facilitating protein assembly. Typically, nuclear-localized lncRNAs interact with transcription factors and regulate gene expression, impacting chromatin organization and transcriptional or post-transcriptional gene control. They are enriched on the chromatin or localized to particular subnuclear compartments and can serve as structural scaffolds of nuclear domains [33, 34].

Aberrant lncRNA expression, notably observed in colorectal tumorigenesis, underscores their significance. Understanding lncRNA functions and their roles in oncogenesis holds promise for CRC prognosis, diagnosis, and treatment development [35, 36].

## Overview of STAT3 pathway

Considerable amounts of signaling pathways related to the carcinogenesis, progression, and metastasis of many types of tumors have been explored. One of the well-established oncogenic signaling pathways is the STAT signal, a transcription factor located in the cytoplasm, which mediates signaling pathways through cellular receptors and participates in numerous cellular activities comprising cell proliferation, cellular survival, differentiation, renewal, immune response, cellular respiration, and immune [37, 38]. STAT protein family includes STAT1, STAT2, STAT3, STAT4, STAT5a, STAT5b, and STAT6. STAT3 is a key member of this family, which is encoded by genes located on chromosome 12 [39]. All these proteins have six common domains in their structure: the oligomerization, coiled-coil domain, Src homology 2 (SH2) domains, DNA-binding domain (DBD), the conserved tyrosine residue (Tyr705 for STAT3), and the transcriptional activation domain [40–42]. STAT3 activation is normally regulated through proteasomal degradation and upstream signaling molecules [43]. Various tyrosine kinases, including receptor tyrosine kinases (RTKs) with intrinsic enzyme activity, including epidermal growth factor (EGFR), platelet-derived growth factor receptor (PDGFR), colony-stimulating factor-1 receptor (CSF1R), and vascular endothelial growth factor receptor (VEGFR) catalyze STAT3 phosphorylation [44]. Nonreceptor tyrosine kinases like Src and abl, in cooperation with cytokine receptors such as IL6R, also contribute to STAT3 tyrosine phosphorylation by activating Janus kinase (JAKs) [45, 46]. JAKs, as a transcription factor, have a vital function in the STAT3 activation. The JAK/STAT pathway includes numerous binding ligands, typically cytokines, for example, interferons (IFNs) and interleukins (ILs), that bind to JAK receptors. Upon the binding, the receptors are dimerized, bringing the receptor-associated JAKs into proximity. After transphosphorylation of JAK, the activated JAKs phosphorylate tyrosine residues in the receptor itself and create binding sites for STAT3 docking. Subsequently, STAT3 binds to phosphorylated tyrosine residues on receptors via its SH2 domain and becomes phosphorylated [47]. Also, some serine kinases, such as NLK, MAPK (p38MAPK, JNK, and ERK), PKC^*δ*,^ and mTOR, can phosphorylate STAT3 in serine position 727, leading to STAT3 maximal transcriptional activity. Moreover, it has been shown that a single lysine residue located at position 685 in STAT3 protein is acetylated by p300 histone acetyltransferase, contributing to the transcriptional activity and homodimer stability of STAT3 [48]. Then, activated STAT3, upon dimerization, enters the nucleus and regulates gene expression by binding to specific DNA sequences in gene promoter regions [49, 50]. It is reported that protein inhibitors of activated STAT (PIAS) and suppressors of cytokine signaling (SOCS) protein families are considered vital negative regulators related to STAT signaling. SOCS can prevent the transduction of the STAT signal through binding and hindering JAKs [51]. PIAS can bind to phosphorylated STAT dimers and stimulate the small ubiquitin-like modifier (SUMO) modification of STAT, resulting in a conformational change or dissociation that prevents STAT3 binding to DNA and its transactivation activity [52]. In CRC, the persistent STAT3 activation results from a lack of upstream regulation. Elevated P-STAT3 levels may be linked to poor prognosis, particularly in CRC and other cancers [53]. Overexpression of STAT3 triggers tumorigenesis and provokes tumor development, metastasis, and recurrence of CRC [54]. Targeting STAT3 prevents in vitro and in vivo tumor growth and metastasis without affecting normal cells, suggesting that STAT3 is a promising molecular target for CRC therapy [55]. One of the correlated mechanisms for the regulation of STAT3 involves ncRNAs such as miRNA and lncRNA. It is reported that they can have a key role in upstream signals and are recognized to participate in the pathogenesis of CRC by triggering or inhibiting the STAT3 signaling cascades. Also, STAT3 can transcriptionally regulate the expression and the activity of various ncRNAs in multiple human cancers, including CRC. To provide new strategies for STAT3-targeted tumor therapy, summarizing the ncRNAs correlated with the STAT3-mediated signaling pathway in CRC is necessary.

## ncRNAs correlated with STAT3 signaling in CRC

### MiRNAs directly target STAT3 in CRC

Recent research has explored the relationship between miRNAs and STAT3 signaling in various cancers, revealing a close correlation. MiRNAs and STAT3 pathways interact through direct and indirect mechanisms, creating feedback loops that impact cellular balance. Understanding these interactions may offer new CRC therapy approaches [56]. MiRNAs act as tumor suppressors by directly targeting and inhibiting STAT3 expression, hindering tumorigenesis. For instance, miR-29a-5p was found to modulate STAT3 protein levels, with increased miR-29a-5p levels in CRC patient colon tissues and CAC mice associated with STAT3 activation. Upregulated miR-29a-5p in vitro led to increased STAT3 expression in colon cancer cells (IEC-6, HCT-116, and RAW264.7). Additionally, IL-6-induced miR-29a-5p upregulation activated STAT3 signaling in CRC cells [57]. Furthermore, Zhao et al. [58] reported that miR-874, a tumor suppressor, is downregulated in CRC, inversely correlating with STAT3 expression. MiR-124, another tumor suppressor, inhibits CRC cell proliferation and induces apoptosis by directly inactivating STAT3 in vitro and in vivo [59]. Yong Wang et al. [60] showed that, in high-grade colon cancer, miR-1299 expression is significantly reduced. MiR-1299 binds to STAT3 3’-UTR, reducing its expression and decreasing proliferation and apoptosis in CRC cells. Furthermore, lower miR-1301 levels promote CRC progression by activating STAT3. MiR-1301 directly targets STAT3, inhibiting cell mobility and invasion in LoVo cells (pSTAT3-positive), suggesting its role as a tumor suppressor by modulating STAT3 in CRC [61].

As mentioned, STAT3 plays a crucial role in maintaining stemness and self-renewal of cancer stem cells (CSCs) in CRC. In HCT-8 CRC cells, it is a direct target of miR-665. Consequently, miR-665 suppression inhibits STAT3 signaling and diminishes the proliferative capacity of CSC and CRC stemness [62]. According to another study, STAT3 is also targeted by miR-296-5p, which exhibits low expression in CRC cells and tissues. Aloperine (ALO), an herbal medicine, elevates miR-296-5p levels, leading to decreased CRC cell proliferation by modulating Bax and Bcl-2 expression, as well as reduced cell migration and invasion by regulating N-cadherin and E-cadherin expression. STAT3 upregulation reverses these effects. ALO inhibits CRC metastasis and proliferation through the miR-296-5p/STAT3 [63].

MiRNAs play a significant role in STAT3-driven tumorigenesis of CRC by regulating cell proliferation, apoptosis, and metastasis. Then, identifying miRNAs that directly target STAT3 may open new insights into developing novel strategies to manage patients with CRC better. All miRNAs directly targeting STAT3 are summarized in Table 1.

**Table 1:** MiRNAs directly target the STAT3 signaling pathway

MicroRNA	Up-/down	Oncogene/suppressor	Biological functions	Target gene	Ref.
**MiR-29a-5p**	Up	Oncogene	Inflammation	STAT3	[57]
**MiR-874**	Down	Suppressor	Cellular growth, apoptosis	STAT3	[58]
**MiR-124**	Down	Suppressor	Cellular proliferation, apoptosis	STAT3	[59]
**MiR-1299**	Down	Suppressor	Proliferation, Apoptosis	STAT3	[60]
**MiR-1301**	Down	Suppressor	Invasion, Migration	STAT3	[61]
**MiR-665**	Down	Suppressor	Stemness	STAT3	[62]
**MiR-296-5p**	Down	Suppressor	Proliferation, migration, invasion	STAT3	[63]

### MiRNAs indirectly regulate STAT3

**Fig. 2:**
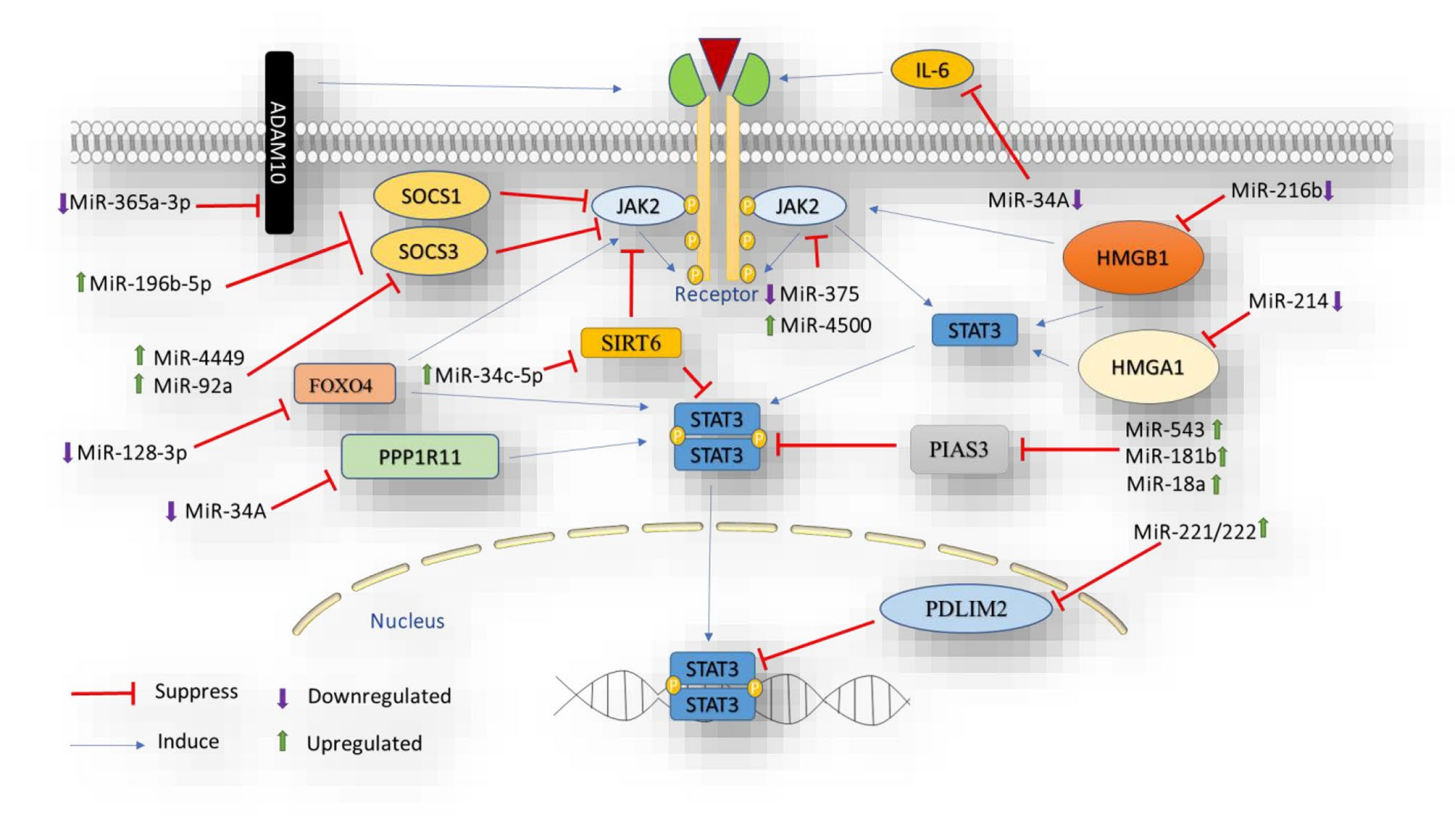
miRNAs involved in indirect regulation of STAT3 signaling

MiRNAs can indirectly regulate STAT3 signaling by targeting other components like IL6/6R, JAKs, SOCS, and PIAS3 **(**Fig. 2**)**. For instance, miR-216b, a tumor suppressor miRNA, targets JAK2/STAT3 signaling via HMGB1 overexpression in CRC. miR-216b directly inhibits HMGB1, and its suppression activates JAK2/STAT3 signaling by restoring HMGB1 expression [64]. Moreover, it has been shown that miR-214 can modulate several intermediate modulators in multiple signaling pathways, including STAT3, by regulating HMGA1 [65]. Previous research has also reported that miR-452, a tumor suppressor miRNA, regulates the JAK1/STAT3 pathway in inflammatory colitis and CRC through IL20RA. In CRC cell lines, miR-452 directly targets IL20RA, resulting in reduced JAK1 and STAT3 levels [66]. PDLIM2, a nuclear ubiquitin E3 ligase involved in STAT3 degradation, is targeted by upregulated miR-221/222 expression. This indirect activation of STAT3 by miR-221/222 via PDLIM2 downregulation promotes cell proliferation and colony formation in CRC cells by reducing STAT3 ubiquitin-mediated degradation through interaction with PDLIM2 mRNA’s 3’UTR [67]. The miR-34 family inhibits EMT and early metastasis in colorectal tumorigenesis. MiR-34 A targets PPP1R11, involved in STAT3 phosphorylation. Ectopic PPP1R11 expression in CRC cells promotes EMT, migration, and invasion through STAT3 signaling. MiR-34 A downregulates PPP1R11, inhibiting STAT3 activation, EMT, and metastasis in CRC cells [68]. MiR-34a participates in the IL-6R/STAT3/miR-34a feedback loop in CRC. IL-6 binds to IL-6R, activating STAT3 and promoting EMT, invasion, and metastasis while downregulating miR-34a. Activated P53 leads to miR-34a-mediated inhibition of IL-6R, suppressing STAT3 signaling [40]. Wu M-Y et al. [69] indicated that IL-6R is also targeted by miR-320 tumor suppressor miRNA in CRC cells. MiR-320, a tumor suppressor, targets IL-6R in CRC cells, inhibiting the IL-6R/STAT3 pathway and preventing colitis-associated CRC in mice models. MiR-196b-5p contributes to CRC cell stemness and chemoresistance to 5-FU by activating and phosphorylating the STAT3 via SOCS1 and SOCS3 targeting [70]. Also, upregulated miR-4449, as an oncogene, promotes CRC cellular proliferation via targeting SOCS3 and subsequent phosphorylation of STAT3. SOCS3, a negative regulator of the STAT3 pathway, is identified as a target gene of miR-4449. Subsequently, inhibition of this miRNA leads to the inactivation of the STAT3 pathway by increasing the expression levels of SOCS3 in CRC cells [71]. Besides, miR-92a has also been reported as an oncomiR that directly binds to SOCS3 and suppresses its expression. Consequently, reduced expression of miR-92a suppressed CRC cells’ stemness by decreasing the spheroid formation ability of these cells and downregulating stemness-related proteins, including p-AKT and p-STAT3, suggesting that the downregulation of p-AKT and p-STAT3-mediated by miR-92a and the suppression of self-renewal and growth in SW480 and LoVo CSC cells could be achieved by SOCS3 overexpression [72]. Suppression of miR-543, as an oncogene, inhibits cell proliferation and metastasis in CRC cells through upregulating PIAS3 expression, preventing the expression of proteins related to proliferation and migration. Remarkably, miR-543 overexpression leads to STAT3 activity, which is reversed through PIAS3 upregulation, showing the importance of miR-543/PIAS3/STAT3 axis in colorectal tumorigenesis [73]. Also, previous research reported that miR-181b also serves as a direct regulator of PIAS3, and its overexpression could downregulate PIAS3, leading to STAT3 activation through phosphorylation in positive feedback in colon cancer cells. This study suggested that miR-181b/PIAS3/STAT3 axis may be a key target for colon cancer therapy regarding its effect on cell growth and metabolism [74]. Wei R et al. illustrated that high-activity miR-375 inhibits CRC cell proliferation by deactivating JAK2/STAT3 and MAP3K8/ERK signaling pathways. However, cellular migration remains unaffected [75]. Ursolic acid increases miR-4500, leading to poly ADP ribose polymerase (PARP) cleavage. Inhibiting miR-4500 suppresses PARP cleavage, enhancing JAK2/STAT3 signaling and apoptosis inhibition in HCT116 CRC cells [76]. Exosomal miR-128-3p suppresses FOXO4, promoting EMT and metastasis in CRC cells by activating JAK/STAT3 and TGF-β/SMAD pathways. Conversely, miR-365a-3p inhibits ADAM10, reducing JAK2/STAT3 phosphorylation, and suppressing proliferation, and metastasis in CRC cells [77, 78]. MiR-21, a well-known oncomiR, promotes colorectal tumorigenesis by suppressing PTEN and activating STAT3 through IL-6, fostering inflammation, invasion, and metastasis. This activation involves the Nf-κB pathway, releasing inflammatory cytokines like IL-1β, IL-6, and TNF-α. IL-6 triggers STAT3 via JAK, forming a positive feedback loop that enhances miR-21 expression [79]. It is also verified that miR-34c-5p stimulated cellular proliferation by regulating the SIRT6-mediated activation of the JAK2/STAT3 signaling pathway in colon cancer cells. It is assumed that the miR-34c-5p/SIRT6/JAK2/STAT3 axis could be an innovative therapeutic target that opens new insights into developing treatment approaches for colon cancer therapy [80].

In conclusion, this section highlights the potential of miRNAs as alternative targets for suppressing STAT3 signaling and its oncogenic consequences in patients with CRC. MiRNAs, though indirectly implicated in STAT3 signaling, exhibit significant promise as oncogenes or tumor suppressors in this context (Table 2**)**.

**Table 2:** MiRNAs indirectly target STAT3

MicroRNA	Up-/down	Oncogene/suppressor	Biological functions	Target gene	Ref.
**MiR-216b**	Down	Suppressor	proliferation, Migration, Invasion, Angiogenesis	HMGB1	[64]
**MiR-214**	Down	Suppressor	Proliferation, Migration, Invasion	HMGA1	[65]
**MiR-452**	Down	Suppressor	Inflammation	IL20RA	[66]
**MiR-221/222**	Up	Oncogene	Proliferation Colony formation	PDLIM2	[67]
**MiR-34 A**	Down	Suppressor	EMT Metastasis	PPP1R11	[68]
**MiR-34a**	Down	Suppressor	EMT Invasion	IL-6	[40]
**MiR-320**	Down	Suppressor	Inflammation Tumor formation Proliferation, Migration, Invasion	IL-6R	[69]
**MiR-196b-5p**	Up	Oncogene	Stemness, Chemoresistance	SOCS1 SOCS3	[70]
**MiR-4449**	Up	Oncogene	Cell proliferation	SOCS3	[71]
**MiR-92a**	Up	Oncogene	Stemness	SOCS3	[72]
**MiR-543**	Up	Oncogene	Proliferation, Metastasis	PIAS3	[73]
**MiR-181b**	Up	Oncogene	Warburg effect growth	PIAS3	[74]
**MiR-375**	Down	Suppressor	Proliferation	JAK2	[75]
**MiR-4500**	Up	Oncogene	Apoptosis	JAK2	[76]
**MiR-128-3p**	Up	Oncogene	Viability, Migration, Invasion, EMT	FOXO4	[77]
**MiR-365a-3p**	Down	Suppressor	Proliferation, Metastasis	ADAM10	[78]
**MiR-21**	Up	Oncogene	Inflammation	PTEN	[79]
**MiR-34c-5p**	Up	Oncogene	Proliferation Apoptosis	SIRT6	[80]
**MiR-18a**	Down	Suppressor	Growth, Proliferation	PIAS3	[81]

### LncRNAs correlated with miRNAs in regulating STAT3

**Fig. 3:**
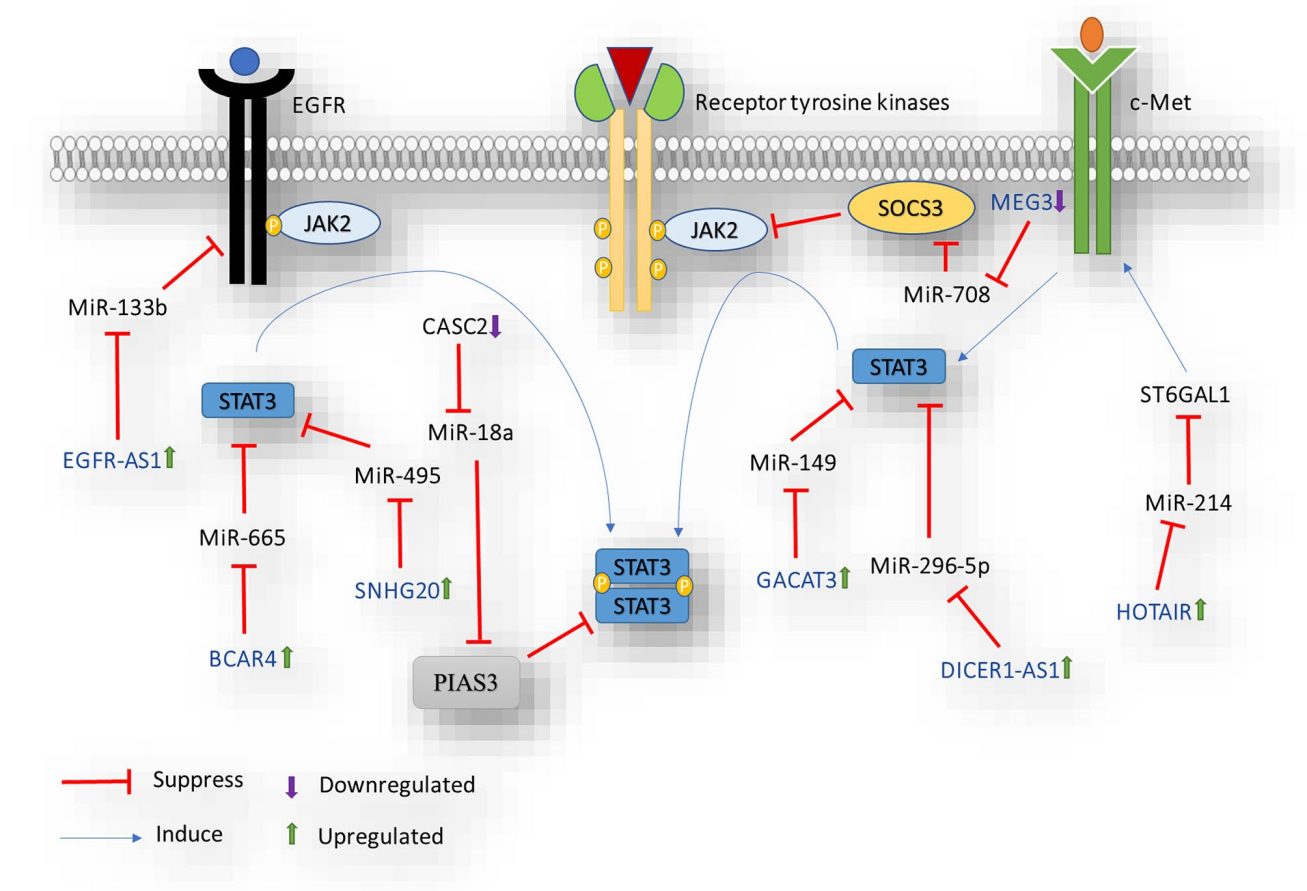
LncRNAs correlated with miRNAs involved in the regulation of STAT3 signaling

Numerous studies confirm an abundance of dysregulated lncRNAs in CRC, highlighting their potential as diagnostic and therapeutic targets. These lncRNAs exert control over diverse signaling pathways and target genes in CRC, often acting as ceRNAs by sequestering miRNAs. (Fig. 3) [35, 82]. In CRC, various dysregulated lncRNAs influence STAT3 signaling, contributing to tumorigenesis. For example, hyper-activated lncRNA EGFR-AS1, as an antisense transcript of EGFR, positively correlates with CRC progression, high EGFR expression level, tumor grade, and metastasis. Also, reduced miR-133b, a direct EGFR target, is inversely associated with elevated EGFR and p-STAT3 in CRC. This suggests EGFR-AS1’s significant role in CRC through miR-133b/EGFR/STAT3 modulation [83, 84]. Oncogenic SNHG20, another lncRNA linked to STAT3, enhances CRC cell proliferation, invasion, and migration via the miR-495/STAT3 axis. Bioinformatics analysis and luciferase reporter assays confirm miR-495 as a direct SNHG20 target and a suppressor of STAT3 activity in CRC. This implies SNHG20’s role in CRC progression by sponging miR-495, resulting in STAT3 upregulation [85]. Additionally, non-coding RNAs’ interaction with STAT3 is vital for CRC stemness. For example, lncRNA BCAR4, which sponges miR-665, sustains stemness and promotes tumorigenicity in CRC cells. LncBCAR4-mediated miR-665 downregulation elevates STAT3 signaling, contributing to oncogenic properties and self-renewal in CRC cells. This underscores the significance of the lncRNA BCAR4/miR-665/STAT3 axis in cancer cell regulation [62]. Tumor-suppressive lncRNA MEG3 regulates STAT3-driven CRC cell stemness by sequestering and downregulating miR-708. In CRC tissue and Apcmin mice, MEG3 downregulation correlates negatively with miR-708, which targets and inhibits SOCS3, a negative JAK/STAT3 signaling regulator. MEG3 overexpression suppresses colonic stem cell proliferation by lowering miR-708 and enhancing SOCS3-mediated STAT3 repression [86]. Moreover, the dysregulated lncRNA GACAT3/miR-149 axis promotes malignant CRC features in vitro and in vivo. GACAT3, abundant in CRC cells and tissues, acts as a miR-149 ceRNA, enhancing oncogenic pathways, including STAT3. Suppressing GACAT3 reduces cellular growth, migration, and invasion in CRC cells and mouse models [87]. Previous research reported that DICER1-AS1 and STAT3 mRNA are upregulated, while miR-296-5p is downregulated in CRC tissues and cell lines, promoting CRC cell growth, migration, invasiveness, and preventing apoptosis. The oncogenic lncRNA DICER1-AS1 regulates the miR-296-5p/STAT3 axis, contributing to CRC carcinogenesis [88]. miR-18a is another miRNA that indirectly activates STAT3 signaling by directly targeting PIAS3. Further investigation indicated that CASC2, as a tumor suppressor lncRNA, could directly regulate PIAS3 expression by acting as a ceRNA for miR-18a, leading to STAT3 inactivation and inhibiting in vitro and in vivo CRC tumor growth [81]. Also, it is reported that aberrant sialylated glycoproteins may result in the progression and development of CRC. A study illustrated that the HOTAIR/miR-214/ST6GAL1 axis is involved in α 2, 6-sialylation of c-met and triggers JAK2/STAT3 signaling pathway. ST6GAL1, a glycosyltransferase family member, induces c-Met sialylation, controlled by HOTAIR via miR-214. Sialylated c-Met enhances JAK2/STAT3 activity, promoting CRC progression [89]. The list of lncRNAs that function as regulators of STAT3-mediated CRC tumorigenesis by modulating the expression of miRNAs is presented in Table 3.

**Table 3:** LncRNAs that participate in the regulation of STAT3 signaling

LncRNA	Up-/down-regulation	Role	Biological functions	Target gene	Ref.
**EGFR-AS1**	Up	Oncogene	Tumorigenesis	miR-133b	[83]
**SNHG20**	Up	Oncogene	Migration Invasion Proliferation	miR-495	[85]
**BCAR4**	Up	Oncogene	Stemness	miR-665	[62]
**GACAT3**	Up	Oncogene	Colony formation Invasion	miR-149	[87]
**DICER1-AS1**	Up	Oncogene	Proliferation, Migration Invasion Apoptosis	miR-296-5p	[88]
**MEG3**	Down	Suppressor	Proliferation	miR-708	[86]
**HOTAIR**	Up	Oncogene	Proliferation, Metastasis Chemoresistance	miR-214	[89]

### LncRNAs directly or indirectly regulate STAT3

LncRNAs also regulate CRC’s STAT3 signaling, impacting components beyond miRNAs. For example, the oncogenic lncRNA AB073614, upregulated in CRC tissue, directly modulates STAT3 phosphorylation, influencing EMT markers like E-cadherin, N-cadherin, Vimentin, and Occludin. AB073614 suppression reduces CRC invasion and migration. The JAK inhibitor AT9283 counteracts AB073614’s effects, suggesting it regulates JAK-STAT3 signaling via JAK-mediated STAT3 phosphorylation [90]. Similarly, highly expressed LINC01106 in CRC tissues predicts patient prognosis. LINC01106 modulates cell proliferation and apoptosis by affecting the STAT3 pathway. LINC01106 Knockdown reduces p-STAT3 and Bcl-2, inhibiting tumor growth [91]. Furthermore, LNRRIL6 regulates the IL-6-mediated STAT3 pathway by binding to the IL-6 gene promoter, leading to increased IL-6 expression and subsequent hyperactivation of the STAT3 pathway. LNRRIL6 upregulation promotes CRC cell survival, proliferation, and in vitro tumor growth through the IL-6/STAT3 axis [92]. Table 4 lists lncRNAs involved in regulating STAT3 signaling, either directly or indirectly.

**Table 4:** LncRNAs directly or indirectly regulate the STAT3 pathway

LncRNA	Up-/down-regulation	Role	Biological functions	Target gene	Ref.
**lncAB073614**	Up	Oncogene	Migration Invasion	JAK	[90]
**LINC01106**	Up	Oncogene	Proliferation Apoptosis	STAT3	[91]
**LNRRIL6**	Up	Oncogene	Proliferation	IL-6	[92]

## STAT3 upregulates or downregulates miRNAs

It has been shown that STAT3 also contributes to the regulation of miRNAs. A study reported that STAT3 suppression using siRNA in SW480 CRC cells leads to either upregulation or downregulation in the expression of some novel miRNAs (Table 5) [54]. According to another study, STAT3 also regulates the expression levels of miR215-5p, miR4521, miR215-3p, and miR30a-5p through colorectal tumorigenesis. The inhibition of STAT3 caused the downregulation of miR215-5p, miR4521, miR215-3p, and the upregulation of miR30a-5p in HT29-derived tumorspheres. Further functional analysis evidenced that STAT3 could suppress the expression of miR30a-5p and hinder apoptosis induction in CRC cells [93]. miR-19a is another miRNA that is transcriptionally upregulated by STAT3 activity in hypoxia. STAT3 transcription factor binds to a region 96 bp/78 bp upstream of miR-19a within its promoter, leading to the induced expression of this oncogenic miRNA. The overexpression of miR-19a was shown to downregulate PTEN mRNA expression, promote cell viability and EMT in CRC cells, and increase tumor growth and metastasis in nude mice models through STAT3/miR-19a/PTEN axis [94]. Moreover, STAT3 has been illustrated to induce the expression of miR-181b as an oncomiR in CRC cells. miR-181b is introduced as a direct regulator of PDCD4, and activating the IL-6/STAT3 signaling pathway increases its expression. This event, in turn, provokes CRC cell proliferation, migration, and apoptosis inhibition by downregulating PDCD4 [95]. Also, STAT3 prompts CRC progression via the novel pathway of miR-572/MOAP-1. MOAP-1 is a proapoptotic protein that participates in apoptosis induction by activation of the Bax protein. It was indicated that miR-572, overexpressed through STAT3 activity, binds to MOAP-1 and induces its downregulation. Consequently, STAT3 induces cell growth, migration, and invasion via miR-572-mediated downregulation of MOAP-1 in CRC cells [96]. In another study, IL-6 has been illustrated to increase miR-92a through IL-6/STAT3 signaling in CRC cells. The findings indicated that STAT3 activated by IL-6 treatment of cells binds to the promoter region of miR-17-92 cluster genes and increases the expression of miR-92a. In turn, miR-92 overexpression was involved in the chemoresistance of CRC cells to 5-FU treatment and induction of stem cell-like features by activating Wnt/β-catenin signaling [97].

Furthermore, STAT3 has also been illustrated to regulate its downstream modulators by regulating some lncRNAs’ expression in tumorigenesis. A report indicated that STAT3 positively modulates the expression of lncRNA DUXAP8 via directly interacting with its promoter. DUXAP8 was shown to be upregulated in CRC tissues, and its suppression diminished cell proliferation, migration, and apoptosis inhibition by upregulating miR-577. This miRNA functions as a tumor suppressor miRNA that targets the RAB14 oncogene. Therefore, it was suggested that STAT3-mediated upregulation of DUXAP8 may participate in CRC tumorigenesis through sponging miR-577 and rescuing RAB14 expression [98].

Moreover, in colon cancer cells, STAT3 was reported to function as an upstream regulator of lncRNA HOTAIR and increase the expression of this oncogenic lncRNA. HOTAIR overexpression, in turn, increased CRC cell invasion through WIF-1 downregulation and subsequent activation of Wnt signaling. In contrast, HOTAIR suppression induced cell apoptosis and hindered the invasion of CRC cells [99]. Additionally, a novel circular ncRNA, circ-STAT3.46, was also reported to be regulated by activation of STAT3 through IGF1/IGF1R/STAT3 pathway. The exposure to IGF-1 could increase circ-STAT3.46 expression in CRC cells, which was reversed through the treatment of cells by AZD1480 JAK2/STAT3 inhibitor. This ncRNA is overexpressed in CRC tissues and positively correlates with the tumor stage. It has also been shown that circ-STAT3.46 sponges and suppresses the expression of miR-139-5p, which in turn rescues miR-139-5p-mediated suppression of IGF1R, leading to activation of STAT3 signaling in colon cancer cells in a feedback [100].

Table 5 indicates the list of miRNAs and lncRNAs regulated by the STAT3 signaling pathway.

**Table 5:** MiRNAs/lncRNAs that are regulated by STAT3

MiRNAs/lncRNA	STAT3 up or downregulates	Ref
MiR-215-5p MiR-4521 MiR-215-3p	Up	[93]
MiR-30a-5p	Down	[93]
MiR-71, MiR-62, MiR-70, MiR-48 MiR-63, MiR-60, MiR-54, Mir-43	Down	[54]
Mir-31 MiR-36 MiR-3 MiR-4 MiR-18 MiR-14 MiR-38 MiR-30 MiR-25	Up	[54]
MiR-19a	Up	[94]
MiR-181b	Up	[95]
MiR-572	Up	[96]
MiR-92a	Up	[97]
circ-STAT3.46	Down	[100]
DUXAP8	Up	[98]
HOTAIR	Up	[99]

## Conclusion and perspective

The STAT3 signaling pathway is a well-known oncogenic pathway that plays a role in cancer formation and progression in a variety of human malignancies, including CRC. Although extensive studies evaluated the molecular mechanisms related to STAT3 in CRC, and many methods were examined to suppress STAT3, eliminating some drawbacks in STAT3 targeted therapies, such as unresponsiveness and resistance, needs further investigation. Clinical trials should be conducted to establish the efficacy and safety of investigational therapeutics targeting STAT3 and its regulating miRNAs and lncRNAs in CRC patients.

This review tried to demonstrate the downstream and upstream regulators of STAT3 in CRC cells in order to enhance understanding of pathways implicated in CRC progression from both therapeutic and diagnostic perspectives. Clinical consequences of these interactions range from diagnosis and prognosis to potential treatment interventions. In CRC, several miRNAs and lncRNAs have been identified as STAT3 negative regulators. They can bind to STAT3 mRNA or alter its expression, hence inhibiting CRC progression. Certain lncRNAs, on the other hand, can promote CRC development by increasing STAT3 activity. These ncRNAs frequently function as oncogenes. (For example, HOTAIR has been found to enhance STAT3 activation and CRC advancement). In fact, miRNAs and lncRNAs, as oncogenes, directly or indirectly activate the STAT3 pathway. In contrast, tumor suppressors ncRNAs prevent the activity of the STAT3 signaling pathway, resulting in the suppression of malignant features of CRC cells. Besides, STAT3 has been shown to exert its oncogenic effects by modulating the expression of multiple regulatory miRNAs and lncRNAs in CRC. Therefore, targeting these molecular regulators could be a promising option for other targeted medicines that are based on the STAT3 signaling pathway. For example, using small interfering RNA (siRNA) as the synthetic mimics of miRNAs technique has increased the hope of successfully treating CRC patients by targeting STAT3 signaling [101]. Aberrant expression of miRNAs and lncRNAs implicated in STAT3 signaling could be used as CRC diagnostic biomarkers. The identification of unique miRNA and lncRNA signatures in patient samples can help with early detection and risk assessment. The deregulation of miRNAs and lncRNAs associated with STAT3 in CRC patients can be suggestive of a poor prognosis. High levels of pro-oncogenic miRNAs and lncRNAs or low levels of tumor-suppressive miRNAs and lncRNAs may be associated with disease progression and poor outcomes. STAT3-mediated interaction between miRNAs and lncRNAs suggests a possible route for CRC therapy. Therapeutic techniques that modify the expression or activity of these miRNAs and lncRNAs may aid in the prevention of CRC progression and metastasis [102]. These methods have a number of drawbacks, including low bioavailability and difficulty in delivering the treatment directly into cancer cells. However, the application of new techniques including nanoparticles that ensure high cellular absorption by encapsulating regulatory RNA molecules and their site-specific administration may open the way for cancer therapy [103].

## Data Availability

Not applicable.
